# Reduced acquisition and reactivation of human papillomavirus infections among older women treated with cryotherapy: results from a randomized trial in South Africa

**DOI:** 10.1186/1741-7015-8-40

**Published:** 2010-06-29

**Authors:** Sylvia Taylor, Chunhui Wang, Thomas C Wright, Lynette Denny, Wei-Yann Tsai, Louise Kuhn

**Affiliations:** 1 Department of Epidemiology, Mailman School of Public Health, Columbia University, New York, NY, USA; 2 Department of Pathology, College of Physicians and Surgeons, Columbia University, New York, NY, USA; 3 Department of Obstetrics and Gynaecology, University of Cape Town, Cape Town, RSA; 4 Department of Biostatistics, Mailman School of Public Health, Columbia University, New York, NY, USA; 5 Gertrude H Sergievsky Center, College of Physicians and Surgeons, Columbia University, New York, NY, USA

## Abstract

**Background:**

Treatment of women for high-grade cervical cancer precursors frequently results in clearance of the associated high-risk human papillomavirus (hrHPV) infection but the role of treatment among women without hrHPV is unknown. We investigated whether cervical cryotherapy reduces newly detected hrHPV infections among HIV-positive and HIV-negative women who were hrHPV negative when treated.

**Methods:**

The impact of cryotherapy on newly detected hrHPV infections was examined among 612 women of known HIV serostatus, aged 35 to 65 years, who were negative for hrHPV DNA, and randomized to either undergo cryotherapy (n = 309) or not (n = 303). All women underwent repeat hrHPV DNA testing 6, 12, 24, and 36 months later.

**Results:**

Among 540 HIV-negative women, cryotherapy was associated with a significant reduction in newly detected hrHPV infections. Women in the cryotherapy group were 55% less likely to have newly detected hrHPV than women in the control group (95% CI 0.28 to 0.71). This association was independent of the influence of changes in sexual behaviors following therapy (adjusted hazards ratio (HR) = 0.49, 95% CI 0.29 to 0.81). Among 72 HIV-positive women, similar reductions were not observed (HR = 1.10, 95% CI 0.53 to 2.29).

**Conclusions:**

Cervical cryotherapy significantly reduced newly detected hrHPV infections among HIV-negative, but not HIV-positive women. These results raise intriguing questions about immunological responses and biological mechanisms underlying the apparent prophylactic benefits of cryotherapy.

## Background

Human papillomavirus (HPV), the causative agent of cervical cancer, is the most common sexually transmitted infection worldwide. Approximately 40% of women become infected with HPV within 2 years of initiating sexual intercourse, and nearly all women are infected at some point in their lifetime [1-5]. A small percentage of women infected with high-risk types of HPV (hrHPV) develop cervical intraepithelial neoplasia (CIN), which can act as a precursor to invasive cervical cancer. Multiple techniques are used to screen for and treat CIN lesions and early stage cervical cancers. Most developed countries have fairly extensive screening programs that primarily utilize cervical cytology as the screening method. Cytology-positive women are usually evaluated with colposcopy and if a high-grade CIN lesion is identified, undergo treatment using a variety of methods that include both excisional and ablative techniques [6]. Unfortunately, this approach to cervical cancer prevention has proven difficult to implement and sustain in many low-resource settings. To address this disparity, novel screen and treat strategies, in which women are screened using a non-cytology-based method and all screen-positive women undergo cryotherapy, have been developed.

One of the more controversial aspects of 'screen and treat' cervical cancer prevention strategies is that a large number of women without high-grade CIN are screen positive and will undergo cryotherapy using this approach. However, there are few data to indicate that cryotherapy of women without high-grade CIN is harmful and there is both a theoretical basis, as well as limited data, to suggest that 'overtreatment' may actually provide marginal beneficial to some patients. It is now widely recognized that the successful treatment of CIN lesions using either excisional or ablative methods frequently results in women becoming hrHPV negative [7]. Although the mechanism responsible for this clearance is unknown, it is presumably mediated by immunological mechanisms as opposed to simple elimination of HPV-infected tissue. However, the effects of treatment on women without lesions are unclear. It is possible that the same mechanisms that cause clearance of hrHPV after cryotherapy in women with high-grade CIN may result in a prophylactic benefit against subsequent acquisition and/or reactivation of hrHPV infections.

The randomized clinical trial of two different screen and treat approaches that we recently conducted in South Africa provided a unique opportunity to evaluate whether or not cryotherapy has a prophylactic benefit for the acquisition/reactivation of hrHPV infections among women free of hrHPV at baseline.

## Methods

### Study design

**Figure 1:**
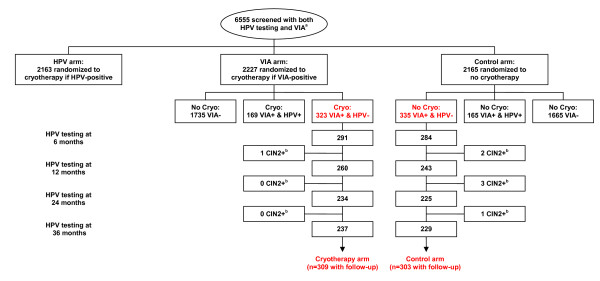
**Flowchart of study design and sampling scheme**. **(a) **All women also received cytology prior to randomization, but only the visual inspection with acetic acid (VIA) result and randomization arm determined whether cryotherapy was performed. **(b) **All women underwent colposcopy/biopsy at 6, 12, 24, and 36 months; if biopsy-confirmed CIN2+ was detected, women were treated with loop electrosurgical excision procedure (LEEP) and exited the study.

This is a secondary analysis of data collected as part of a randomized clinical trial assessing the safety and efficacy of screen and treat [8]. The primary analyses have been published previously [8]. The current analysis describes hrHPV acquisition/reactivation over a 36-month period of time among a cohort of 612 women who were hrHPV negative and who were randomized to either undergo cryotherapy or to be followed without treatment unless a CIN 2,3 lesion was identified. This analysis was possible since the larger trial evaluated two different screen and treat strategies. In one arm all HPV positive women underwent cryotherapy. In the other arm, all visual inspection with acetic acid (VIA) positive women underwent cryotherapy. The third arm of the trial consisted of a control group of women, none of whom, regardless of their HPV or VIA test results, received cryotherapy. Therefore, we can compare hrHPV negative women in the VIA-based arm who were VIA positive and underwent cryotherapy (n = 309) with similar hrHPV negative women in the control arm who also were VIA positive but did not undergo cryotherapy (n = 303) (Figure 1).

### Study population

All participants were previously unscreened women, aged 35 to 65 years, recruited through community education and outreach activities focused on cervical cancer prevention in Khayelitsha, Cape Town, South Africa between June 2000 and December 2002. All participants provided written informed consent and the study was approved by the Institutional Review Boards of Columbia University and the University of Cape Town.

### Study procedures and follow-up

All women had cervical samples collected for hrHPV testing and underwent the VIA screening test at baseline prior to randomization. HrHPV testing used the Hybrid Capture 2 DNA assay (Digene Corporation, Gaitherburg, MD, USA) and probes for 13 'high carcinogenic risk' HPV types as previously described [8]. Samples were considered positive for high-risk HPV DNA if ≥ 1 pg HPV DNA/ml was detected. A positive VIA test was defined as any acetowhite lesion and no attempt was made to differentiate the acetowhitening of metaplasia from cervical intraepithelial neoplasia. Cervical specimens were also obtained for molecular testing for *Neisseria gonorrhoeae *and *Chlamydia trachomatis *(Hybrid Capture for CT/GC, Digene). Liquid-based cervical cytology (ThinPrep Pap Test, Cytyc Corporation, MA, USA) was also performed. All women received counseling for confidential HIV serotesting and anonymous HIV serotesting at each visit. HIV serotesting was performed using the Abbott HIV 1/2 g 0 Kit on the Abbott AXSYM system (Abbott, Chicago, IL, USA); positive results were confirmed using the Vironsticka HIV uniform 2 plus 0 kit (Organon Teknika, Durham, NC, USA). Questionnaires, including sociodemographic, clinical and sexual behavior questions, were administered at each visit.

Women were scheduled for a return visit 2-6 days after the initial examination and, if eligible for randomization, were randomized to one of the three groups. Women randomized to receive cryotherapy had the treatment provided by a trained nurse using N_2_O_2 _and a standard cryosurgical unit (Wallach Surgical Devices, Orange, CT, USA) using a 3-min freeze, thaw, and second 3-min freeze. A complete description of the initial examination procedures and randomization schema has been published previously [8].

After treatment, women were instructed to abstain from vaginal intercourse, douching or using any intravaginal products for 4 weeks and given both male and female condoms to use in the event they did not abstain. Women were asked to return to the clinic 4 weeks later, at which time a questionnaire was administered that focused on potential side effects and complications of cryotherapy and recent sexual activity. All women included in the current analysis were asked to return to the clinic at 6, 12, 24, and 36 months. At these visits hrHPV testing was repeated and all women underwent a colposcopic exam with endocervical curettage and biopsy of all acetowhite lesions. Clinicians performing the colposcopy were blinded to all HPV and cytology results. All women with CIN grade 2 or greater (CIN 2+) detected on biopsy at any visit were recalled and treated with loop electrosurgical excision procedure (LEEP) and exited the study [8].

### Statistical analysis

Kaplan-Meier methods were used to describe the cumulative probability of testing positive for high-risk HPV DNA among women in the two groups [9]. A log-rank test was used to compare survival curves between the groups. Analyses were stratified by HIV status ascertained over 36 months since HPV acquisition/reactivation rates are known to differ substantially by HIV status [10,11]. In an attempt to distinguish reactivated from newly acquired infections, we stratified by any reported sexual activity during the month prior to enrolment or at 1 or 6 months after enrolment. Sexual activity during the month prior to initial enrolment was taken into consideration because the time from initial exposure to HPV and active production of virus is approximately 3 weeks [12]. Sexual activity beyond 6 months could not be taken into consideration because detailed sexual histories were not collected at 12, 24, and 36 months.

Cox regression models were fitted to evaluate the effect of cryotherapy on HPV acquisition/reactivation, using a discrete method for treatment of ties [9], and hazard ratios (HRs) were reported to measure the association. Age, *C. trachomatis*/*N. gonorrhoeae *infection at enrolment, age at first sexual intercourse, and the lifetime number of sexual partners were examined as potential confounders. In addition, as women were encouraged to refrain from sexual activity or condom use for 4 weeks following treatment, and sexual activity is strongly associated with HPV acquisition, variables for sexual activity and condom use at 4 weeks and 6 months were included in the multivariate model. These analyses were also stratified by HIV serostatus. We also performed these analyses only among women with a normal baseline cytological diagnosis in order to exclude those with potentially false negative HPV results at baseline.

## Results

Of the 658 women who were hrHPV negative eligible for this analysis, 612 (93%) had HPV results available for at least 1 follow-up visit and were included in the analysis. Among the HIV-negative women (n = 271 in the cryotherapy arm and 269 in the control arm), none of the baseline characteristics differed significantly by study arm (Table 1). Among the HIV-positive women (n = 38 in the cryotherapy arm and n = 34 in the control arm), those in the cryotherapy arm were significantly less likely than controls to report ≥ 5 sexual partners in their lifetime (45% vs 71%, *P *= 0.027), but there was no significant difference in the reported number of sexual partners during the month prior to enrolment (3% ≥ 2 in both arms), marital status (34% vs 35% married), or any other baseline characteristic.

**Table 1:** Baseline sociodemographic characteristics and risk factors for cervical disease at enrolment

	No. of subjects (%)			
	
	HIV negative		HIV positive	
	
	Cryotherapy arm, (n = 271)	Control arm, (n = 269)	Cryotherapy arm, (n = 38)	Control arm, (n = 34)
Age, years:				
35-39	110 (40.6)	114 (42.4)	19 (50.0)	21 (61.8)
40-49	124 (45.8)	116 (43.1)	18 (47.4)	9 (26.5)
50-65	37 (13.7)	39 (14.5)	1 (2.6)	4 (11.8)
Education:				
No school	28 (10.3)	28 (10.4)	3 (7.9)	3 (8.8)
Some primary school	103 (38.0)	98 (36.4)	13 (34.2)	11 (32.4)
Some high school	108 (39.9)	103 (38.3)	17 (44.7)	13 (38.2)
High school graduate	32 (11.8)	40 (14.9)	5 (13.2)	7 (20.6)
Currently employed	73 (26.9)	70 (26.0)	10 (26.3)	3 (8.8)
Married	141 (52.0)	150 (55.8)	13 (34.2)	12 (35.3)
Age < 16 years at first sexual intercourse	98 (36.2)	102 (37.9)	12 (31.6)	13 (38.2)
≥ 5 Lifetime sex partners	84 (31.0)	82 (30.5)	17 (44.7)^a^	24 (70.6)^a^
≥ 2 Sex partners during previous month	3 (1.1)	4 (1.5)	1 (2.6)	1 (2.9)
Current smoker	21 (7.8)	19 (7.1)	6 (15.8)	4 (11.8)
Current contraceptive use:				
Injectable	57 (21.0)	44 (16.4)	10 (26.3)	9 (26.5)
Oral	10 (3.7)	5 (1.9)	0	2 (5.9)
No. of live births:				
None	5 (1.9)	10 (3.7)	0	3 (8.8)
1-4	181 (66.8)	184 (68.4)	29 (76.3)	24 (70.6)
≥ 5	85 (31.4)	75 (27.9)	9 (23.7)	7 (20.6)
Cytologic abnormality (ASCUS or greater)	15 (5.5)	20 (7.4)	2 (5.3)	3 (8.8)
*Chlamydia trachomatis *or *Neisseria gonorrhoeae*	16 (5.9)	13 (4.8)	6 (15.8)	2 (5.9)
*Trichomonas vaginalis*	30 (11.1)	25 (9.3)	3 (7.9)	4 (11.8)

^a^*P *value < 0.05 for comparisons between the cryotherapy and control group.

ASCUS = atypical squamous cells of undetermined significance.

**Figure 2:**
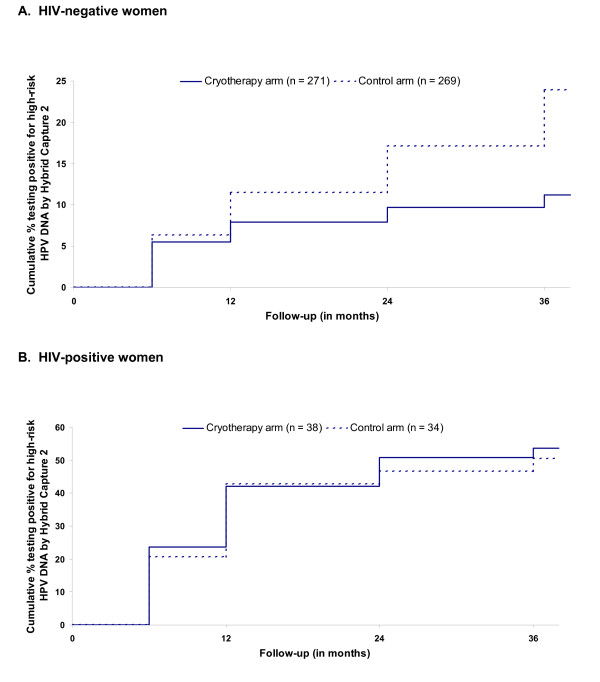
**Human papillomavirus (HPV) acquisition/reactivation**. HPV acquisition/reactivation among cryotherapy-treated and untreated women who tested negative for high-risk HPV DNA but were visual inspection with acetic acid (VIA) positive at baseline.

Overall, among HIV-negative women, cryotherapy was associated with a substantial reduction in the rate of newly detected HPV infections (*P *= 0.0004), and the crude HR was 0.45 (95% CI 0.28 to 0.71) (Figure 2a, Table 2). The rates of newly detected HPV infections only diverged between the two groups after 6 months of follow-up and by 36 months, the cumulative rate of new detection of HPV infection among women in the cryotherapy arm was 11.2% versus 24.8% in the control arm. Among HIV-positive women, there was no difference in newly detected HPV infection in those receiving cryotherapy at any time point. After 36 months the cumulative newly detected high-risk HPV infection rates was 53.7% versus 50.5% (HR = 1.10, 95% CI 0.53 to 2.29) (Figure 2b, Table 2). The associations between cryotherapy and newly detected HPV infection were significantly different by HIV status (*P*_interaction _= 0.044).

**Table 2:** Cumulative probability of human papillomavirus (HPV) acquisition/reactivation among HIV-positive and HIV-negative women with and without cryotherapy

	Cumulative no. with a newly detected HPV infection (cumulative%)				
		
	6 months	12 months	24 months	36 months	*P *value for log-rank test
HIV-negative women					
Total sample:					
Cryotherapy arm (n = 271)	15 (5.5)	21 (7.9)	25 (9.7)	28 (11.2)	0.0004
Control arm (n = 269)	17 (6.3)	30 (11.5)	45 (18.0)	59 (24.8)	
Sexual activity reported during 1 month prior to enrolment or at 1 or 6 months after enrollment:					
Cryotherapy arm (n = 238)^a^	13 (5.5)	18 (7.7)	22 (9.7)	25 (11.3)	0.002
Control arm (n = 236)^b^	16 (6.8)	27 (11.8)	42 (19.4)	56 (27.2)	
No sexual activity reported during 1 month prior to enrollment and at 1 and 6 months after enrollment:					
Cryotherapy arm (n = 31)^a^	2 (6.5)	3 (10.4)	3 (10.4)	3 (10.4)	0.96
Control arm (n = 29)^b^	1 (3.5)	3 (10.3)	3 (10.3)	3 (10.3)	
HIV-positive women					
Total sample:					
Cryotherapy arm (n = 38)	9 (23.7)	16 (42.1)	19 (50.8)	20 (53.7)	0.8
Control arm (n = 34)^c^	7 (20.6)	14 (42.8)	15 (46.6)	16 (50.5)	
Sexual activity reported during 1 month prior to enrollment or at 1 or 6 months after enrollment:					
Cryotherapy arm (n = 33)	8 (24.2)	12 (36.4)	15 (46.4)	16 (49.8)	0.65
Control arm (n = 29)^c^	7 (24.1)	14 (49.4)	15 (54.0)	15 (54.0)	
No sexual activity reported during 1 month prior to enrollment and at 1 and 6 months after enrollment:					
Cryotherapy arm (n = 5)	1 (20.0)	4 (80.0)	4 (80.0)	4 (80.0)	0.08
Control arm (n = 3)	0	0	0	0	

^a^Excludes two women who could not be classified as either sexual active or inactive during the first 6 months of the study.

^b^Excludes four women who could not be classified as either sexual active or inactive during the first 6 months of the study.

^c^Excludes two women who could not be classified as either sexual active or inactive during the first 6 months of the study.

In an attempt to distinguish reactivation from a newly acquired infection, analyses were stratified by either any reported sexual activity 1 month before baseline, or at 1 and 6 months after baseline (Table 2). As more than 90% of the study sample reported sexual activity during this time period, results for sexually active women were similar to those for the total sample. However, among non-sexually active, HIV-negative women (31 in cryotherapy group and 29 in the control group), cryotherapy was not associated with reduced HPV (*P *= 0.96). Although this group is quite small, it is the group in which most newly detected HPV infections are likely to represent reactivation of a previously acquired infection. There were too few sexually inactive HIV-positive (five in the cryotherapy group and three in the control group) women to evaluate the impact of sexual activity.

To further explore the robustness of the observed protective effect of cryotherapy in HIV-negative women, we performed stratified analyses by age, marital status, number of lifetime sexual partners, sexual activity at baseline, and sexual activity at 1 month following cryotherapy. The protective effect was consistent in every stratum (HR ranged from 0.31 to 0.55), except among women older than 50 years (HR = 0.98, 95% CI 0.29 to 3.28) (Table 3). After adjusting for age, age at first sexual intercourse, number of life time sexual partners, *C. trachomatis*/*N. gonorrhoeae *infection at enrolment, sexual behaviors at months 1 and 6 after enrolment, cryotherapy was still associated with a 51% reduction in HPV acquisition/reactivation rate among HIV-negative women (adjusted HR = 0.49, 95% CI 0.29 to 0.81), but not among HIV-infected women (adjusted HR = 1.06, 95% CI 0.35 to 3.18).

**Table 3:** Overall effect of cryotherapy on human papillomavirus (HPV) acquisition/reactivation and stratified analyses among HIV-negative women

	Hazard ratio (95% CI) (N = 540)
Crude hazard ratio (HR)*	0.45 (0.28 to 0.71)
Stratified analyses	
Age:	
35-39	0.42 (0.20 to 0.91)
40-49	0.38 (0.19 to 0.73)
50-65	0.98 (0.29 to 3.28)
Marital status:	
Married	0.55 (0.29 to 1.04)
Not married	0.35 (0.18 to 0.69)
Lifetime sexual partners:	
≥ 5	0.31 (0.11 to 0.86)
< 5	0.50 (0.30 to 0.83)
Sexual activities	
At baseline (1 month prior to baseline):	
Not active	0.44 (0.17 to 1.15)
Sexually active	0.45 (0.27 to 0.76)
In first month (1 month after baseline):	
Not sexually active	0.38 (0.19 to 0.78)
Sexually active	0.48 (0.26 to 0.88)

*The adjusted HR = 0.49 (0.29 to 0.81), adjusted for age, age at first sex, lifetime sex partners, sex and condom use at months 1 and 6.

## Discussion

In this screen and treat trial, cryotherapy was associated with a 50% reduction in the probability of newly detected hrHPV infection over a 3-year period among women who were hrHPV negative when treated. This magnitude of reduction in HPV acquisition/reactivation is similar to the protective benefits observed among sexually active women in the randomized trials of the prophylactic HPV 16/18 vaccines [13,14]. These results raise intriguing questions about the possible mechanisms underlying the prophylactic benefits of cryotherapy.

A potential confounder to the inference that cryotherapy is responsible for the reduced rates of hrHPV infection would be a change in sexual behavior after cryotherapy. In this study, women undergoing cryotherapy were encouraged to refrain from sexual intercourse or, if intercourse occurred, to use condoms for at least 4 weeks following treatment. However, our analyses suggest that the protective effect of cryotherapy on acquisition/recurrence of hrHPV is unlikely to be explained by a change in sexual behavior. Since an impact of cryotherapy on newly detected HPV infections was not observed at the first visit (6 months) post randomization and only emerged after 12 months of follow-up, it is unlikely that acute changes in sexual behavior post cryotherapy are responsible. Moreover, the stratified analyses showed that cryotherapy produced the same protective effect regardless of a woman's reported sexual behavior. After adjusting for sexual behaviors at 1 month and/or 6 months post randomization, as well as condom use, cryotherapy was still associated with a decreased risk of HPV acquisition/reactivation among HIV-negative women (adjusted HR = 0.49, 95% CI 0.29 to 0.81).

Protective immunity against HPV is poorly understood, but is thought to result from the interplay of non-specific innate immunity and antigen-specific adaptive immunity [15]. HPV utilizes a number of strategies to avoid evoking the principal innate immunity danger signals. The virus only expresses non-secreted proteins at a low level and there is no viremia and limited antigens for systemic presentation [15]. HPV infection also downregulates major histocompatibility complex (MHC) class I in the epithelium and cytolysis of HPV-infected keratinocytes does not lead to inflammation [16]. We speculate that cellular injury produced by cryotherapy could induce a cascade of immunological responses by upregulating various cytokines that mediate the innate, cellular, and humoral immune responses [17]. An adaptive antigen-specific immunity could then develop following this non-specific innate immunity stimulation. Alternatively, non-specific innate immunity evoked by cryotherapy-induced injury might result in clearance or enhanced suppression of latent hrHPV infections and produce the observed effects. Furthermore, if the prophylactic effects of cryotherapy on hrHPV acquisition/reactivation are a result of stimulation of host immune responses, this might explain why no effect was observed in HIV-positive women. It is well established that HIV infection increases the likelihood of anogenital HPV infections and that the increased risk is primarily attributable to the immunological effects of HIV infection [10,11,18]. Not only have hrHPV acquisition/reactivation rates been shown to be greatest in HIV-infected women with low CD4+ cell counts, but HIV infection is also associated with impaired local mucosal immune response to HPV, including downregulation of cytokines and suppression of proinflammatory and anti-inflammatory responses [19].

Alternatively, cryotherapy may reduce HPV acquisition by reducing cervical ectopy [20,21], which is highly associated with HPV infection and may specifically increase the risk of infection with HPV types in the α9 clade, such as HPV16 [22-24]. Further, by targeting the cervical transformation zone, where lesions usually arise [25], cryotherapy may directly destroy cells harboring latent infections.

This study has several potential limitations. It was not possible to determine whether cryotherapy reduced HPV acquisition or reactivation or both since molecular tests for hrHPV do not distinguish between the two and nearly all of the women in our study reported past and current sexual activity. In the small subset of women reporting no sexual activity, there was discernible benefit of cryotherapy on what we presume to be reactivations, but inference is limited due to the relatively small number of women in this group. Further, the few infections identified in this group could potentially be accounted for by false negative baseline results since the hrHPV assay that we used detects only relatively high copy numbers of HPV [26]. We also do not have detailed data on specific HPV genotypes to determine if they were differentially affected. Misclassification of sexual activity or HPV acquisition through other transmission modes (for example, hand to genital contact) may also have occurred.

Our study included only a relatively small number of HIV-positive women. Since most of these women reported sexual activity we could not distinguish HPV acquisition from reactivation. HIV-infected women are known to be at high risk of reactivating established infections [10,11], thus, a larger sample size may be needed to detect significant reductions in acquisition.

Our trial design provided a unique opportunity to evaluate the prophylactic impact of cryotherapy on hrHPV acquisition/reactivation in women who did not have detectable hrHPV DNA when treated. Prior reports on the effects of various cervical treatment modalities on hrHPV acquisition/reactivation have been limited by the fact that treated women had biopsy-confirmed CIN and were presumably persistently infected with at least one hrHPV at the time of treatment. Moreover, since most women with high-grade CIN undergo treatment, it has been difficult to find appropriate control groups to determine the impact of therapy of hrHPV acquisition/reactivation [27]. One recent publication from the ALTS prospective follow-up trial compared new HPV infections in HPV-positive women who underwent LEEP and those who did not [27]. This study found that treatment of CIN 2,3 with LEEP had little impact on acquisition of HPV during subsequent 6-month and 21-month periods. However, there was a suggestion that treatment might slightly reduce the acquisition of hrHPV types, especially those of the α9 clade. Although our randomized clinical trial was not specifically designed to address the subgroup of women that we report on here, the study randomization resulted in two large and comparable groups, strengthening our inferences that the unexpected observation may reveal a true biological effect.

## Conclusions

Our results suggest that cryotherapy may produce a prophylactic benefit, protecting women against hrHPV acquisition/reactivation. We hypothesize that this protective effect is due to the activation of innate or adaptive immune responses following cryotherapy-induced injuries but immunological studies would be needed to test this hypothesis and other mechanisms may be involved. Irrespective of the mechanism of action, our current findings demonstrating a protective effect of cryotherapy on acquisition/reactivation of hrHPV combined with our previous findings that cryotherapy reduces the incidence of CIN 2,3 in hrHPV positive women [8] strongly supports the promotion of HPV-based screen and treat cervical cancer prevention strategies. These strategies not only treat prevalent disease but may also reduce the risk of developing disease over the long term which is an important benefit especially in populations without access to routine, repeat cervical cancer screening.

## Competing interests

LD has participated in various speaker roles for GlaxoSmithKline, and Merck & Company. TCW is a consultant to Gen Probe, Roche Molecular Diagnostics, GlaxoSmithKline, and Merck & Company. The other authors have no conflicts to declare.

## Authors' contributions

ST conducted the original analysis and wrote the first draft. CW, LK and W-YT conducted analyses and gave statistical input. TCW, LD and LK designed the study, collected data and revised the manuscript.

## Pre-publication history

The pre-publication history for this paper can be accessed here:

http://www.biomedcentral.com/1741-7015/8/40/prepub
